# The Multifunctional Role of Chitosan in Horticultural Crops; A Review

**DOI:** 10.3390/molecules23040872

**Published:** 2018-04-10

**Authors:** Rahat Sharif, Muhammad Mujtaba, Mati Ur Rahman, Abdullah Shalmani, Husain Ahmad, Toheed Anwar, Deng Tianchan, Xiping Wang

**Affiliations:** 1 College of Horticulture, Northwest A&F University, Yangling 712100, China; rahatsharif2016@nwafu.edu.cn (R.S.); husain@nwafu.edu.cn (H.A.); 2 Institute of Biotechnology, Ankara University, Ankara 06110, Turkey; mujtaba@ankara.edu.tr; 3 State Key Laboratory of Crop Stress Biology in Arid Areas, College of Horticulture, Northwest A&F University, Yangling 712100, China; mati89@yahoo.com; 4 State Key Laboratory of Crop Stress Biology in Arid Areas, College of Life sciences, Northwest A&F University, Yangling 712100, China; abdullqadir36@yahoo.com; 5 Hubei Collaborative Innovation Center for Grain Industry/Research Center of Crop Stresses Resistance Technologies, Yangtze University, Jingzhou 434025, China; toheed.agric92@yahoo.com; 6 School of Mechanical Aerospace and Civil Engineering, The University of Manchester, Manchester M13 9PL, UK; cindydeng1234@163.com; 7 Key Laboratory of Horticultural Plant Biology and Germplasm Innovation in Northwest China, Ministry of Agriculture, Northwest A&F University, Yangling 712100, China

**Keywords:** biopolymer, vegetables, fruits, biotic stress

## Abstract

Chitosan is a naturally occurring compound and is commercially produced from seafood shells. It has been utilized in the induction of the defense system in both pre and post-harvest fruits and vegetables against fungi, bacteria, viruses, and other abiotic stresses. In addition to that, chitosan effectively improves the physiological properties of plants and also enhances the shelf life of post-harvest produces. Moreover, chitosan treatment regulates several genes in plants, particularly the activation of plant defense signaling pathways. That includes the elicitation of phytoalexins and pathogenesis-related (PR) protein. Besides that, chitosan has been employed in soil as a plant nutrient and has shown great efficacy in combination with other industrial fertilizers without affecting the soil’s beneficial microbes. Furthermore, it is helpful in reducing the fertilizer losses due to its coating ability, which is important in keeping the environmental pollution under check. Based on exhibiting such excellent properties, there is a striking interest in using chitosan biopolymers in agriculture systems. Therefore, our current review has been centered upon the multiple roles of chitosan in horticultural crops that could be useful in future crop improvement programs.

## 1. Introduction

Recently, chitosan has been one of the most preferred biopolymers due to its biocompatibility, antioxidant, anticancer, biodegradability, antimicrobial, and non-toxic properties as well as being an economical material, produced from waste resources such as seafood shells [1,2,3]. Structurally, chitosan is a linear polymer, composed of two sub-units as d-glucosamine and N-acetyl-d-glucosamine, linked with each other through 1,4-glycosidic bonds [4,5]. Since the last decade, chitosan research is increasing due to its significant diverse uses in several fields of life i.e., plant sciences [6,7,8,9] and medical sciences [10]. About 8700 chitosan-related records are available on Scopus. On a commercial scale, chitin has been extracted by following a classic, well-known acid-base method. The samples are demineralized using acids followed by a deproteinization with a base, respectively [6,7,8]. However, the functionality of chitin increases when it is converted into chitosan, a derivative form of chitin [9]. The presence of amine groups on chitosan makes it prone to structural modifications resulting in a functional derivative of chitin [10]. Besides agriculture, chitosan has a large set of applications, such as in the food, cosmetic, textile, and biomedical industries [11,12,13,14].

In plants, the chitosan is largely used to mimic biotic and abiotic stresses. The first study of using chitosan as an antipathogen in plants was reported by Allan and Hadwiger [15], where they demonstrated the fungicidal effects of chitosan on different cell wall compositions of fungi. The improvement of the defense system after applying chitin and chitosan, both in monocotyledon and dicotyledons is the center of addressing this biopolymer in multi-research area [16]. Chitosan has been a bio-fungicide, bio-bactericide, and bio-virucide, which spurs plant defense system against the pathogen, thus inducing the immune system of plants, fruits, and vegetables [12,15,17,18,19]. Furthermore, the growing demand for food also stimulated the increased use of industrial fertilizer, which causes serious environmental unbalance and is having catastrophic effects on human health. Therefore, the use of chitosan as a biofertilizer is considered. Chitosan has been reported to have a positive effect on rhizobacteria growth, where Chitosan possesses a symbiotic relation with growth promoting rhizobacteria, thus triggered germination rate and improving plant nutrient uptake [20].

This review presents the plethora of research conducted on the uses of chitosan in horticulture crops. In particular, we present the utilization of chitosan in coping with biotic and abiotic stresses, improving the physiological attributes of plants, post-harvest quality of fruits and vegetables, and reducing the use of inorganic fertilizer.

## 2. Sources of Chitosan

Chitosan is the *N*-deacetylated derivative of chitin. It is a natural polysaccharide, which can be produced after the alkaline deacetylation of chitin (essential structural polymer, constituting a large fraction of insects and a crustacean’s exoskeleton) [21,22,23,24]. Briefly, the chitin extracted isolates are refluxed in 60% NaOH solution (*w*/*v* 1:15 or 1:20, where *w* = mass of chitin, *v* = volume of NaOH solution) for 3–4 h by stirring at a temperature of 80–100 °C. In order to neutralize the pH level of the isolated chitosan, the samples are washed with deionized, distilled water. Then, the chitosan samples are dried for 24 h in an oven using a temperature of 60–80 °C and the chitosan percentage is then calculated. Below we compiled different sources of chitin and chitosan (Table 1).

## 3. Structure and Characterization of Chitosan

**Figure 1:**
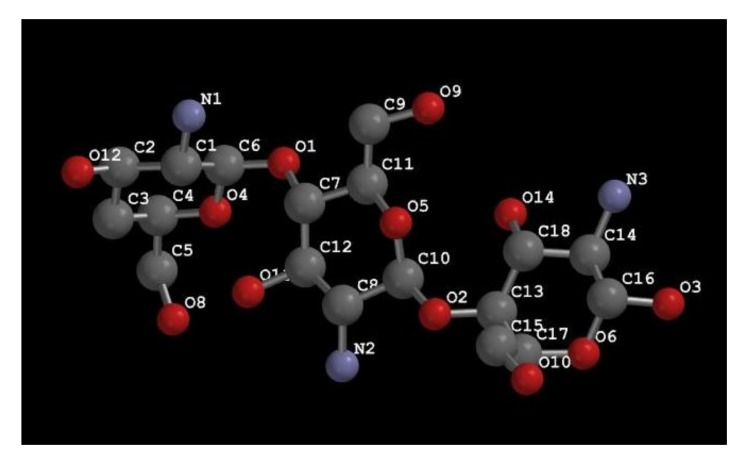
Structural design and chemistry of chitosan.

Chemically, chitosan is a linear polymer composed of two sub-units, d-glucosamine and *N*-acetyl-d-glucosamine, linked with each other through 1,4-glycosidic bonds [4,5]. The general structure of the chitosan molecule is made up of three rings (Figure 1). Chitosan exhibits three functional groups, primary and secondary hydroxyl groups and amine groups [45]. Due to these functional groups, they can easily undergo chemical modification. In addition, these functional groups affect the solubility and mechanical properties of chitosan. Chitosan also has beta-1, 4 glycosidic linkages. The oxygen atoms (O1 and O2) are attached to the C6–C7 and C10–C13 atoms. Compare to chitin, chitosan is more soluble in acidic aqueous mediums. The solubility comes from the protonation of –NH_2_ at the C-2 position of the d-glucosamine repeat unit which induces the conversion of the polysaccharide to a polyelectrolyte in acidic media [5]. The solubility character of chitosan broadens its scope of applications in almost every field of man’s life and health (agriculture, medical, process engineering, industries etc.). In literature reports, the structural chemistry of chitosan has been vastly characterized using different analytical tools, including FT-IR, NMR, and XRD. These techniques are actively being applying for the identification and detailed characterization of chitosan obtained from different sources, such as honeybee [6], *Vespa crabro* (wasp) [7], *Leptinotarsa decemlineata* [21], *Daphnia longispina* (Crustacea) [22], *Asellus aquaticus*, *Agabus bipustulatus*, *Anax imperator*, *Hydrophilus piceus*, *Notonecta glauca*, *Ranatra linearis* [42], Orthoptera species (*Calliptamus barbarus* and *Oedaleus decorus*) *Ceriodaphnia quadrangula* [46,47,48,49]. The FT-IR peaks for chitosan are known to be around 1650 cm^−1^ and 1590 cm^−1^ which correspond to amide I band (carbonyl ν(C=O)) and amide II (amine ν(NH_2_) tensions), respectively [46,48]. Like chitin, chitosan also degrades in two stages. In the first stage, 2–4% of mass loss can be recorded at the temperature ranging from 0 to 150 °C. This first stage of degradation can be ascribed to the evaporation of the bound water molecules in the chitin structure. The second degradation stage can be recorded between the temperature ranges of 150–650 °C, causing mass loss of around 60–90%. The maximum thermal stability peak (°C) of chitosan varies from source to source but on average, the thermal stability of chitosan ranged between 280–310 °C [21]. 

Chitosan exhibits several reactive amino side groups which enhance the applicability of chitosan and offer the possibility of formation of a large variety of chitosan derivatives.

Chitooligosaccharides (COS) or oligochitosan is one of the several useful water-soluble derivatives of chitosan. Like all other polysaccharides, chitosan can also be cleaved by hydrolyzing agents due to the unstable glycosidic bonds. COS can be produced by different methods such as acid hydrolysis [47], enzymatic hydrolysis [49], oxidative degradation [50], and ultrasonic degradation [51]. *N*-carboxymethyl chitosan (CM-chitosan) is a water-soluble derivative of chitosan which has many applications in the food and medical industries, and in gene therapy [52,53]. *N*-carboxymethyl chitosan can be prepared by treating chitosan with glyoxylic acid. Chitosan is water-soluble at pH > 7, however at pH 2.5–6.5  phase separation can be seen because of the equilibrium between charges (positive and negative) on the polymer. The excess of charges (positive or negative) determines the solubility of CM-chitosan [33]. Trimethylchitosan ammonium is another water-soluble cationic derivative of chitosan. It is produced by quaternization of chitosan i.e., reacting low acetyl content chitosan with methyl iodide and sodium hydroxide. Trimethylchitosan ammonium displays flocculating properties such as kaolin dispersions, making it applicable in paper manufacturing [33]. *N*-methylene phosphonic chitosan (NMPC) is an anionic derivative, exhibiting amphoteric properties. NMPC possesses bonding efficiency for cations such as Ca^2+^ and many transition metals (Cu (II), Cd (II), Zn (II)) [54].

Lactic-Glycolic Acid-Chitosan hydrogels, another functional derivative of chitosan, showed a stronger interaction between water and chitosan chains after grafting with lactic and/or glycolic acid. It can be produced by direct grafting of D,L-lactic and/or glycolic acid on chitosan without any catalysts. It has potential applications in biomedical applications including drug delivery systems and wound dressings (134).

## 4. Antimicrobial Activity of Chitosan

**Figure 2:**
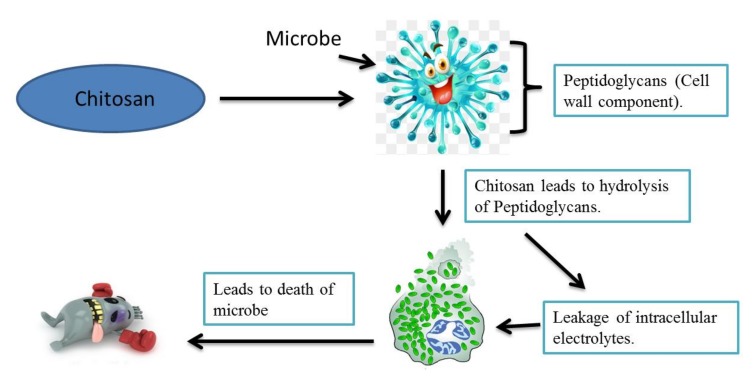
Chitosan acts on the pathogen by breaking the peptidoglycan bond and causing intracellular electrolyte leakage which leads to the death of the microbes.

The increase in the number of research papers related to chitosan’s antimicrobial activity prove it as a versatile biostimulant in the horticulture sector. The elicitation of the defense system in both pre- and post-harvest fruits and vegetables is highly promising. Therefore, regarding the antimicrobial mechanism of chitosan, several researchers have presented their practical point of view. For example, Goy et al. suggested three antibacterial mechanisms of chitosan; firstly, ionic surface interaction resulting in cell wall leakage; secondly, permeation of chitosan into microorganism nuclei inhibits their protein and mRNA synthesis, and thirdly, formation of an external film over the plant surface, limiting the nutrient availability for microorganisms [55]. Liang et al. [56] stated that chitosan is responsible for the destruction of the bacterial cell membrane which causes death due to the leakage of intracellular substances [56]. It was also found to be involved in altering the growth of fungi and reduced toxin production [57]. However, in recent times, it has been reported that chitosan is responsible for the hydrolysis of peptidoglycans (cell wall component), increasing electrolyte leakage and potentially causing the death of the pathogen (Figure 2) [58]. 

### 4.1. Chitosan Effect on Fungi, Bacteria, and Nematodes

Since 1979, when chitosan was reported as a bio fungicide by Allan and Hadwiger [15], it has attracted an ample amount of attention in terms of research in the field of plant protection. In addition, it is also well documented in controlling plant disease caused by bacteria. However, in nematodes, not a great amount of research material is available and therefore further study is required to assess the role of chitosan as a potential nematocide. The effectiveness of chitosan against different pests and pathogens has covered a large number of research articles across different fruits and vegetable. Therefore, from the big collection of chitosan research, we enlisted recent studies (Table 2) reporting the fungicidal, bactericidal, and nematocidal capacity of chitosan in various fruits and vegetables. This body of research suggests that chitosan can be use a bio-stimulant of the plant immune system to tackle diverse unfriendly environments.

### 4.2. Effect of Chitosan on Viral Diseases

The induction of plant resistance against viruses has been reported widely [75]. Generally, viruses that affect plants systematically are more threatening. Therefore, utilization of chitosan as a virucide was reported as the most feasible approach to limit viral infection [76]. Chirkov et al. [77] reported that application of chitosan on potato plants infected with potato virus X (PVX) showed resistance to PVX virus. Moreover, tomato plants treated with chitosan not only showed resistance to tomato mosaic virus but also their vegetative growth was improved [78]. Similarly, chitosan in formulation with plant growth promoting rhizobacteria (PGRP) conferred resistance to leaf curl virus in tomato plant [79]. Also, chitosan was found effective against *squash mosaic virus* (SMV) [80,81]. Furthermore, Chirkov et al. [18] hypothesized that there might be some peculiar properties of the host plant which initiate the antiviral reaction(s) after chitosan treatment. In line with that, chitosan oligosaccharide induces resistance against *tobacco mosaic virus* (TMV) by activation of the salicylic acid signaling pathway [82].

## 5. Chitosan as an Insecticide

Chitosan shows strong resistance to microbial diseases and insecticidal activity against various plant pests. However, chitosan derivatives have been found to be potentially more harmful than the pest. Due to these reasons, more chitosan derivatives have been developed in the recent past [83]. In line with that, chitin derivative (*N*-2-chloro-6-fluorobenzyl-chitosan) was found to be lethal against the oleander aphid (*Aphis nerii*) and larvae of leaf-worm (*Spodoptera littoralis*) of cotton crops [83]. In addition, nano-chitosan (CSg PAA) was also identified as a potential insecticide against the insects of soybean i.e., *Aphis gossypii* and *Callosobruchus maculatus*, as it significantly reduces the number of eggs deposited by the female [84]. More recently, a new chitosan derivative named Avermectin-grafted-*N*,*O*-carboxymethyl chitosan (NOCC) was obtained and has shown excellent insecticidal activity against armyworms, carmine spider mites, black bean aphids, and brown plant hoppers [85]. Despite having a strong killing impact against the above-mentioned insects, it might have the same impact on its family members as well. According to the reported studies, the above mentioned insects are involved in causing irreversible damage to tomato [86,87], cucumber [88], eggplant, potato, and chili [89]. Moreover, some insects exhibit chitin in their exoskeleton which can result in resistance to chitosan-based insecticides. To overcome such problems, chitosan has been fed to reared carnivorous insects in order to use them as biological controllers of chitinous pests [90]. Therefore, it would valuable to utilize chitosan as a bio-insecticide for horticultural crops.

## 6. Chitosan’s Effects on Abiotic Stresses

### 6.1. Effect on Drought Stress

Drought stress is one of the most important multidimensional environmental stressors that damage plants’ physiology, biochemical properties, and molecular traits [91]. For example, in apples, young seedlings were foliar sprayed with chitosan, which enhanced antioxidant activity, reduced electrolyte leakage, and restored moisture content under continuous drought stress for 35 days [92]. It was also reported to induce resistance against drought stress in potato, moth orchid, rice, white clover, and grapevine by means of induced antioxidant activities, increased endogenous H_2_O_2_ content, enhanced endogenous chitosan activities, and root system development [93,94,95,96,97]. Moreover, chitosan induced ABA activity, which plays a key role in the regulation of stomatal aperture and reduced the rate of transpiration when the plant is going through stress phase [98,99]. Therefore, we suggest that chitosan might be a potential antitranspirant that helps horticulture crops cope with drought stress.

### 6.2. Effect on Heat Stress 

Heat stress is often a complicated issue in agricultural species as it usually occurs simultaneously with drought stress. This makes it hard for the plant researcher to distinguish between the two stresses [100]. It has been reported that in dry bean production under heat stress, chitosan treatment could be the best approach to escape heat stress in late-sown plants [101]. To the best of our knowledge, there is not a large amount of research available on chitosan involvement in response to heat stress. However, the participation of ABA in coping with heat stress [102] via its involvement in inducing HS-related genes is reported in many published notes [103,104]. Overexpression of ABF3 (abscisic acid responsive element-binding factor 3) confers tolerance to heat stress [104]. Therefore, the use of chitosan might be effective for overcoming high-temperature stress by triggering ABA activity, which further induces the expression of defense-related ABA-responsive genes in horticultural species.

## 7. Chitosan Effects on Fruits and Vegetables

### 7.1. Chitosan Effects on Plant Growth, Yield Attributes, and Physiological Activities

The chitosan effects on fruit physiology and agronomic traits have been highly studied using different concentrations of chitosan over a variety of fruits. In line with that, foliar spraying of 5 mL L^−1^ chitosan over mango trees improved the number of fruits tree^−1^, weight and size of fruit, and vegetative growth [105]. In grapes, chitosan was sprayed at 500 L per hectare during the pre-bunch closure and veraison stage and effectively increased the POX and PAL activities, polyphenol content, and SOD activities [106,107,108]. Moreover, an increase in the weight of fresh fruit of the kiwi plant was observed after spraying with chitosan in field conditions [109]. Furtherly, Gayed et al. reported that chitosan in combination with calcium chloride reduced the early swelling of peach trees, maintained freshness and firmness of fruits, and decreased the weight loss percentage [110]. In nectarine, chitosan improved the soluble solid content and also helped maintained the post-harvest firmness of the fruit [111].

Like in fruits, chitosan also positively affects the agronomic traits of vegetables. Tomato plants were subjected to chitosan treatment, resulting in high phenolic compound and PPO activities, production of phytoalexins, and improvement in fruit weight and overall yield [71,112,113]. Tsugita et al. suggested that application of chitosan in daikon radish triggered the growth of roots and shoots [114]. Similarly, it was reported in sweet basil, grapevine, *Gerbera*, *Dendrobium* orchids, and in cabbage, that chitosan treated plants showed better growth than that of controls [115,116,117,118,119]. Moreover, the cucumber plant is highly susceptible to low-temperature stress. However, when treated with chitosan, the plants had reduced reactive oxygen species, improved photosynthetic capacity, and the membrane system was strengthened to alleviate cold stress [120]. In chili, seeds treated with chitosan had significantly improved germination rates, germination index, mean germination time, and germination after accelerated aging, as well as high seed quality and enhanced storage life [121]. Furthermore, we also enlisted some crops on which the impact of chitosan was highly effective in terms of enhancing agronomic traits (Table 3). In the context of the above discussion, chitosan use is recommended to enhance the photosynthetic activity, vegetative growth, antioxidant activities, fruit quality attributes, and overall growth and yield of the crop.

### 7.2. Chitosan Effects on Post-Harvest Fruits and Vegetables

The diverse beneficial effects of chitosan in fruit shows great value. Furthermore, a coating of post-harvest mango with edible chitosan showed a reduction in the percentage of rotten tissue, increased ascorbic acid content, increased shelf-life, and maintained freshness [134,135,136]. Likewise, in pomegranate, edible chitosan coating enhanced the shelf life and improved freshness for up to 16 days with good chemical and sensory characteristics during post-harvest cold storage, and kept the surface microbial growth under check [137,138]. Petriccione et al. [139] reported upregulated anthocyanins activity, delay in color changes, and preservation of water content in chitosan-coated sweet cherries [139]. Similarly, in post-harvested strawberries, chitosan significantly prolonged the anthocyanins, polyphenol, and antioxidant activities, while inhibiting flesh browning under cold storage conditions [140]. Furthermore, thin-peel rose apples showed a reduction in disease severity, sustained fruit firmness, and a reduction in the weight loss percentage during post-harvest storage when treated with 2% chitosan [141]. The significance of chitosan under cold storage has also been reported for post-harvest apricots, where it has shown improvement in antioxidant enzyme activities and elevating the total phenolic content [142]. Bananas are a highly perishable fruit and deteriorate faster than other fruits. Edible chitosan can be used to delay the ripening and to enhance the shelf life of bananas. Further, it also improves the antioxidant activity and vitamin C content [143]. Green mold disease has a substantial effect during post-harvest storage and transportation of citrus. Coating with edible chitosan maintained fruit firmness, surface color, juice content, and other quality attributes [144]. Ripening and fruit senescence in peach is a major issue that affects their economic value. However, treatment with chitosan resulted in a significant increase in antioxidant enzyme activity, senescence arrest, delayed fruit ripeness, and maintenance of shape and color [70,110]. In kiwi fruit, the high molecular weight of chitosan was found to be effective in increasing the shelf life, fruit firmness, and other quality parameters [145]. It is clear from the above references that chitosan is involved in improving the physical structure and shelf life and maintaining the quality of post-harvest stored fruits. 

Refrigeration of tomatoes during summer causes a considerable amount of loss. However, use of chitosan significantly enhanced the shelf life, visual appearance, and other quality attributes of the refrigerated tomatoes [146]. Likewise in carrot, edible chitosan was applied to carrot slices, which reduced the ripening process, reduced sugar, and increased the total phenolic content [147]. As in broccoli, chitosan treatment along with mild heat shock improved the shelf life and also maintained the sensory attributes [148]. Further, chitosan was used to induce chill tolerance, enhanced shelf life, preserve fruit quality, and improve antioxidant activities in post-harvest cucumber and cantaloupe melon [149,150]. Nowadays, people are more concerned about food safety and quality, and due to this reason chitosan has potential to be utilized in different methods in order to enhance the innate immunity of post-harvest products. 

## 8. Chitosan Effects on Gene Expression

Gene expression is a biological process which changes according to changing environment, and allows the cells to respond to environmental stimuli. Different exogenous gene regulators have been reported in recent studies. Here, we present some of the recent research done on chitosan involvement in the regulation of gene expression. Hadwiger [151] reported that chitosan activates genes and inhibits RNA synthesis in fungi. The chitosan activates several genes and increases production of proteins and phenolic compounds through the phenylpropanoid pathway, which in turn increases tolerance against pathogens [151]. In *Dendrobium,* an ornamental flowering plant, chitosan treatmentdown-regulated the expression of the *ycf2* gene in young leaves, conferring enlarged chloroplasts [152]. This suggests the important role of chitosan in increasing the flowering ability of ornamental plants through gene pathway regulation. In addition, Hadwiger stated that PR genes are induced by chitosan [153]. Chitinase and a β-1,3 glucanase, two PR proteins, contribute to plant protection against fungal infection by degrading the fungal cell wall [154]. In tomato plants, chitosan extracts have been used to alleviate the effect of two tomato pathogens, namely *Alternaria solani* and *Xanthomonas vesicatoria*. The result suggested that chitosan significantly upregulated the expression of the *PINII* marker, which is responsible for activating the defense signaling pathways [155]. Similarly, in pepper, raspberry, and strawberry plants, chitosan was found to be effective in inducing plant defense mechanisms due to the higher expression level of chitinase (EC 3.2.1.14) and *β-1,3 glucanase* (EC 3.2.1.39) genes [156,157,158,159]. Moreover, in grapes, the plants pre-treated with chitosan from three different commercially-produced formulations showed higher endochitinase activity and two of the chitosan formulations triggered exo-chitinase activity [160]. However, there is still a lack of research examining the role of chitosan treatment in PR genes under different stresses. Furthermore, the *MLO* clade V genes in vegetables are a gateway for the pathogen of powdery mildew disease, which has been confirmed in the recent report by Berg et al., to restore the susceptibility to powdery mildew in cucumber [161]. In contrast to that, chitosan is effective against widespread fungal diseases. Therefore, research is required to evaluate the impact of chitosan on *MLO* clade V gene expression and how much it affects the disease severity index. Below, we tabulated (Table 4) some of the recent studies demonstrating the role of Chitosan in regulating gene expression.

## 9. Chitosan as a Bio-Fertilizer and Fertilizer Protectant

Ecological toxicity is at a critical point due to the high-level production and usage of inorganic fertilizers. Therefore, biodegradable biofertilizers, like chitosan, are attracting the research community to avoid the hazards of using inorganic fertilizers. Chitosan gets degraded enzymatically without affecting the soil-borne beneficial rhizosphere biota at low concentrations, and also induces the symbiotic exchange between plant and microbes [73]. In addition, chitosan is a polysaccharide-based biopolymer, which stimulates the activity of plant symbiotic microbes, resulting in the alteration of rhizosphere microbial equilibrium, thus disadvantaging the plant pathogens [173,174]. In recent research, chitosan was used as a biofertilizer to improve crop yield with less environmental contamination. In line with that statement, chitosan in combination with lysozyme has shown beneficial effects, where it significantly reduced the rate of lesions in tomato stems to 14% [175]. In potato, late blight is an important disease that causes economic damage to potato yields. However, after soil inoculation with chitosan as a biofertilizer, a significant reduction in tuber infestation by late blight was detected, a significant increase in plant nutrient uptake was also recorded. The study was done to produce organic potato seeds for an organic grower [176]. Similarly, 1% chitosan mixed with fertilizer improved the nitrogen and phosphorous content in the roots and shoots of *Eustoma grandiflorum* (Raf) compared with non-chitosan mixed soil grown plants [177]. In Chinese cabbage, plants treated with a chitin-based product showed faster growth than plants treated with a standard mineral fertilizer [178]. In another study, chitosan in combination with N, P, K fertilizer proved to be significant in altering the effect of *Botrytis cinerea*-caused grey mold disease in *Begonia* × *hiemalis* Fotsch, and also increased the antioxidant activities and other commercial traits [179]. Besides that, soil supplemented with chitin enhanced plant growth by improving nutrient uptake [180]. Additionally, inoculation of soil with 0.1% chitin from crustaceans induces defense against root-infecting fungi in chili plants and also improved the nutrient uptake [181]. Likewise, NPKB bio-fertilizer mixed with the chitosan-containing fungi *Cunninghamella elegans* produced NPKP influenced melon plant growth positively and also improved NPK uptake [182]. Furthermore, tomato plants irrigated with chitosan exhibited decreased multiplication of root-knot nematodes and *Pochonia chlamydosporia* (a parasitic fungi), resulting in improved root length, weight, and increase shoot growth (Nuria Escudero et al., 2017) [73]. Furthermore, *Ornithogalum saundersiae* bulbs coated with chitosan produced earlier flowers and had broader leaves (Salachna et al., 2015) [183]. In another report by Salachna et al. 2017, chitooligosaccharide in combination with gellan gum induced vigorous growth, high antioxidant activity, and increased polyphenol content of *Ornithogalum saundersiae* [184]. This highly suggests its role as a potential bio-fertilizer. However, the use of chitosan as a bio-fertilizer in horticulture crops is quite underestimated and not a great amount of research material is available on it. Therefore, it might be useful to use chitosan bio-fertilizer over different horticulture crops to evaluate its effect on growth parameters against different stresses. Furthermore, chitosan bio-fertilizers are available commercially from different manufacturers. CANADA OCEANIC sells it in the name of *Softguard*, as a potential biofertilizer that can be used for a variety of purposes (www.canadaoceanic.com/Fertilizers/SoftGuard.shtml).

The other important aspect of using chitosan is to control the release of fertilizer. It is estimated that about 40–70% of nitrogen, 80–90% of phosphorus, and 50–70% of potassium from applied fertilizers is lost to the environment and cannot be absorbed by plants [185]. Due to this, fertilizers are overused with horticultural crops, and vegetables in particular are highly affected by fertilizer toxicity. Nitrate is also considered as an important parameter of water pollution due to its involvement in surface and groundwater contamination [186]. Similarly, application of excessive nitrogen leads to water contamination, as surplus nitrogen is usually carried away by water or leaches down to pollute the water table [187]. Therefore, to minimize the negative effects of agriculture on the surrounding environment, it is important to reduce the use of fertilizer [188]. Chitosan has been used recently to control the release of inorganic fertilizers in order to limit the harmful effects of excessive fertilization [189,190,191]. Wu et al. [189] suggested that the use of chitosan coating on fertilizer improves the efficiency of plant fertilizer uptake and also reduce the production cost. Therefore, in order to minimize the overuse of chemical fertilizers in vegetables and fruit by reducing the leaching and volatilization process, local farmers must consider chitosan as a coating material, according to the suggested model of Hähndel [192].

## 10. Conclusions

The plethora of available and ongoing research on chitosan utilization continues to present its efficacy. Chitosan has shown great importance in improving the physiological mechanisms and post-harvest shelf life of fruits and vegetable against biotic and abiotic stress. In addition, chitosan derivatives possess good insecticidal activity, however, to the best of our knowledge, there is no report on chitosan use in a field study against insects in major horticultural crops. Besides that, chitosan plays a role in regulating gene expression and inducing molecular defense systems in plants and post-harvest produce. Moreover, chitosan bio-fertilizer and fertilizer coated in chitosan triggers the plant growth more, compared to synthetic fertilizers. Furthermore, chitosan and its derivatives provide good antifungal and nematocidal activity without disturbing the beneficial microbes in the soil and can be considered as a green approach for soil sterilization [73,193]. Therefore, more research is required to utilize chitosan against heat stress, for the application of chitosan to the root zone, to prevent nematode-borne diseases, and to reduce the overuse of synthetic fertilizers in horticulture crops.

## Figures and Tables

**Table 1:** Sources of chitin and chitosan from various aquatic and terrestrial organisms.

Aquatic	Terrestrial	Microorganisms
Crustaceans Crab *Chionoecetes opilio* [25] *Podophthalmus vigil* [26] *Paralithodes amtschaticus* [27] *Carcinus mediterraneus* [28]	Arthropods Spiders *Geolycosa vultuosa* [29] *Hogna radiate* [29] *Nephila edulis* [30]	Fungi (cell walls) Ascomydes *Mucom rouxii* [31] Blastomycota Blastocladiaceae [32] Chytridiomycota Chytridiaceae [33] Protista Brown algae [34] Planta Green algae [34]
	Scorpionxs *Mesobuthus gibbosus* [35]	
Water lobster Crayfish [36]		
Prawn *Aristens antennatus* [37]	Beetles *Bombyx mori* [38] *Holotrichia parallela* [39] *Leptinotarsa decemlineata* [38] Cockroaches [40]	
Krill *Daphnia longispina* [22] *Anax imperator* [41] *Hydrophilus piceus* [41] *Notonecta glauca* [41] *Agabus bipustulatus* [41] *Asellus aquaticus* [41]		
	Brachiopods *Lingula seta* [42]	
Mollusca Squid pens *Loligo sp* [43] *Todarodes pacificus* [44]		
Coelenterata		

**Table 2:** Defense mechanisms induced by chitosan in horticultural crops.

Crop	Concentration	Pathogen/Pest	Defense Mechanism	Mode of Application	References
Banana	1.0% (*w*/*v*)	*Anthracnose*	Arresting fungal activity	In-vivo	[59]
Carrots	2 or 4% (*w*/*v*)	*Sclerotinia sclerotiorum*	Antifungal activity	In Vitro	[60]
Cucumber	0.2 g L^−1^	*Botrytis cinerea*	Antifungal	Foliar spray	[61]
Cucumber	2% (*w*/*v*)	*Sphaerotheca fuliginea*	Antifungal	Petri dish treatment	[62]
Chilli pepper	0.32% (*w*/*v*)	*Colletotrichum capsici*	Hijacked fungal activity	In vivo	[63]
Eggplant	20 mL	*Ralstonia solanacearum*	Reduce fungal caused wilt	Cotton leaf disk elicitation method	[64]
Mango	1% (*w*/*v*)	*Colletotrichum gloeosporioides*	Fungus inhibition	Post-harvest coating	[65]
Orange	2% (*w*/*v*)	*Penicillium italicum* and *Penicillium digitatum*	Fungicidal effect	Post-harvest coating	[66]
Pear	25 g/L	*A. kikuchiana* and *P. piricola*	Antifungal activity	Post-harvest treatment	[67]
Papaya	1.5% (*w*/*v*)	*C. gloeosporioides*	Fungicidal effect	In situ	[68]
Palm	1 mg mL^−1^	*Fusarium oxysporum*	Inhibition of root fungal activity	Soil inoculation	[69]
Peach	0.5 g L^−1^	*Monilinia fructicola*	Antioxidant and antifungal	Dipping in solution	[70]
Tomato	1 mg/mL	*Alternaria solani*	Antibacterial defense	Foliar application	[71]
Tomato	0.1% (*w*/*v*)	*Fusarium oxysporum f. sp. Lycopersici*	Antifungal	Foliar application	[72]
Tomato	0.1 mg mL^−1^	*Pochonia chlamydosporia*	Nematocidal effect	Fertigation	[73]
Tomato	10 mg L^−1^	*R. solanacearum*	Antibacterial	Seed treatment	[74]

**Table 3:** Reported studies on chitosan effects on the agronomic traits of horticultural crops.

Crop	Functions	Reference
**Artichoke**	Improved seed germination and plant growth	[122]
**Chili**	Leaf area, canopy diameter and plant height	[123]
**Cucumber**	Triggered vegetative growth and quality of cucumber fruits	[124]
**Coffee**	Plant height and leaf area	[125]
**Chinese cabbage**	Uniform seed germination and enhanced seedling growth	[126]
***Dendrobium formosum* orchid**	Enhanced seed germination	[127]
**Eggplant**	Improved antioxidant activity and total phenolic content	[64]
**Grapevine**	Increased number of internode and improved rooting	[97]
**Okra**	Plant height, leaf number and fruit yield	[128]
**Potato**	Increased the fresh weight of tuber and overall yield	[129]
**Peach**	Induced antioxidant activity and defense-related enzymes	[70]
**Radish**	Enhanced the nutrient uptake efficiency and mimic cadmium stress	[130]
**Strawberry**	Produced fruits with an increased shelf life	[131]
**Tomato**	Improved fruit and productivity	[71,72]
**Tea**	Enhanced the phenolic content up to 9%	[132]
**Watermelon**	Increased in weight of fresh and dry seedlings, Stimulated the growth of the primary stems, the root system, and an increase in stomatal width.	[19,133]

**Table 4:** Involvement of chitosan in regulating gene expression.

Crop	Genes	Expression	Functions	Reference
Arabidopsis	*PAD3*	↑	Induced resistance against *Botrytis cinerea*	[162]
*Capsicum annuum*	*cat1*, *pal*, *pr1*	↑	Maintain plant fitness and keep the defense system alert for the upcoming stress.	[163]
Dendrobium officinale	*DoWRKY1*	↑	Proper regulation of plant metabolic and physiological properties.	[164]
Ginger	*GLU*, *PAL*	↑	Triggered tolerance against rhizome rot caused by *Fusarium oxysporum*	[165]
Mango	*POD*	↑	Altered the severity of anthracnose disease by inducing enzymatic activity.	[166]
Peach	*POD*, *GLU*	↑	Enhanced resistance against brown rot	[70]
Satsuma Orange	*CHI*, *PAL*	↑	Inhibiting the fungal decay process in orange fruit caused *Penicillium digitatum*.	[167]
*Sweet orange*	*Cellulose synthase*	↓	Reduce cell wall growth and increase resistance against biotic stress.	[168]
Strawberry	*Fra a1*, *Fra a3*, *Fra a4*	↑	Fruit development, flavonoid biosynthesis and fruit ripening	[169,170]
Scrophularia striata Boiss	*PAL*	↑	Improved antioxidant activities and increased the production of phenylpropanoid	[171]
Tomato	*MPK3*, * MPK6*, *PR1a1*	↑	Increase resistance against grey mold caused decay via the activation of MAPK signaling pathway.	[172]

↑ showing up-regulation and ↓ down-regulation of the respective genes presented in the table.

## References

[1] Dash M., Chiellini F., Ottenbrite R., Chiellini E. (2011). Chitosan—A versatile semi-synthetic polymer in biomedical applications. Prog. Polym. Sci..

[2] Shukla S.K., Mishra A.K., Arotiba O.A., Mamba B.B. (2013). Chitosan-based nanomaterials: A state-of-the-art review. Int. J. Biol. Macromol..

[3] Vroman I., Tighzert L. (2009). Biodegradable polymers. Materials.

[4] Muzzarelli R.A. (1973). Natural chelating polymers; alginic acid, chitin and chitosan. Natural Chelating Polymers; Alginic Acid, Chitin and Chitosan.

[5] Rinaudo M. (2006). Chitin and chitosan: Properties and applications. Prog. Polym. Sci..

[6] Kaya M., Mujtaba M., Bulut E., Akyuz B., Zelencova L., Sofi K. (2015). Fluctuation in physicochemical properties of chitins extracted from different body parts of honeybee. Carbohydr. Polym..

[7] Kaya M., Sofi K., Sargin I., Mujtaba M. (2016). Changes in physicochemical properties of chitin at developmental stages (larvae, pupa and adult) of Vespa crabro (wasp). Carbohydr. Polym..

[8] Kaya M., Bitim B., Mujtaba M., Koyuncu T. (2015). Surface morphology of chitin highly related with the isolated body part of butterfly (Argynnis pandora). Int. J. Biol. Macromol..

[9] Rinaudo M. (2008). Main properties and current applications of some polysaccharides as biomaterials. Polym. Int..

[10] Shamov M., Bratskaya S.Y., Avramenko V. (2002). Interaction of carboxylic acids with chitosan: Effect of pK and hydrocarbon chain length. J. Colloid Interface Sci..

[11] Cervera M.F., Heinämäki J., de la Paz N., López O., Maunu S.L., Virtanen T., Hatanpää T., Antikainen O., Nogueira A., Fundora J. (2011). Effects of spray drying on physicochemical properties of chitosan acid salts. AAPS PharmSciTech.

[12] Kaya M., Akyuz L., Sargin I., Mujtaba M., Salaberria A.M., Labidi J., Cakmak Y.S., Koc B., Baran T., Ceter T. (2017). Incorporation of sporopollenin enhances acid–base durability, hydrophobicity, and mechanical, antifungal and antioxidant properties of chitosan films. J. Ind. Eng. Chem..

[13] Akyuz L., Kaya M., Koc B., Mujtaba M., Ilk S., Labidi J., Salaberria A.M., Cakmak Y.S., Yildiz A. (2017). Diatomite as a novel composite ingredient for chitosan film with enhanced physicochemical properties. Int. J. Biol. Macromol..

[14] Mujtaba M., Salaberria A.M., Andres M.A., Kaya M., Gunyakti A., Labidi J. (2017). Utilization of flax (Linum usitatissimum) cellulose nanocrystals as reinforcing material for chitosan films. Int. J. Biol. Macromol..

[15] Allan C.R., Hadwiger L.A. (1979). The fungicidal effect of chitosan on fungi of varying cell wall composition. Exp. Mycol..

[16] Barber M., Bertram R., Ride J. (1989). Chitin oligosaccharides elicit lignification in wounded wheat leaves. Physiol. Mol. Plant Pathol..

[17] Chaouat C. (2013). Conception de Nouveaux Systèmes de Formulation d'actifs Dépigmentants, en vue de leur Utilisation par voie Cutanée.

[18] Chirkov S.N., Surguchova N., Atabekov J.G. (1994). Chitosan inhibits systemic infections caused by DNA-containing plant viruses. Arch. Phytopathol. Plant Prot..

[19] Li B., Shi Y., Shan C., Zhou Q., Ibrahim M., Wang Y., Wu G., Li H., Xie G., Sun G. (2013). Effect of chitosan solution on the inhibition of Acidovorax citrulli causing bacterial fruit blotch of watermelon. J. Sci. Food Agric..

[20] Agbodjato N.A., Noumavo P.A., Adjanohoun A., Agbessi L., Baba-Moussa L. (2016). Synergistic effects of plant growth promoting rhizobacteria and chitosan on in vitro seeds germination, greenhouse growth, and nutrient uptake of maize (*Zea mays* L.). Biotechnol. Res. Int..

[21] Kaya M., Baran T., Erdoğan S., Menteş A., Özüsağlam M.A., Çakmak Y.S. (2014). Physicochemical comparison of chitin and chitosan obtained from larvae and adult Colorado potato beetle (*Leptinotarsa decemlineata*). Mater. Sci. Eng. C.

[22] Kaya M., Cakmak Y.S., Baran T., Asan-Ozusaglam M., Mentes A., Tozak K.O. (2014). New chitin, chitosan, and O-carboxymethyl chitosan sources from resting eggs of Daphnia longispina (Crustacea); with physicochemical characterization, and antimicrobial and antioxidant activities. Biotechnol. Bioprocess Eng..

[23] Kumar M.R., Muzzarelli R.A., Muzzarelli C., Sashiwa H., Domb A. (2004). Chitosan chemistry and pharmaceutical perspectives. Chem. Rev..

[24] Rabea E.I., Badawy M.E.-T., Stevens C.V., Smagghe G., Steurbaut W. (2003). Chitosan as antimicrobial agent: Applications and mode of action. Biomacromolecules.

[25] Crespo M.P., Martínez M.V., Hernández J.L., Yusty M.L. (2006). High-performance liquid chromatographic determination of chitin in the snow crab, Chionoecetes opilio. J. Chromatogr. A.

[26] Das S., Ganesh E.A. (2010). Extraction of chitin from trash crabs (*Podophthalmus vigil*) by an eccentric method. Curr. Res. Biol. Sci..

[27] Sperstad S.V., Haug T., Paulsen V., Rode T.M., Strandskog G., Solem S.T., Styrvold O.B., Stensvåg K. (2009). Characterization of crustins from the hemocytes of the spider crab, Hyas araneus, and the red king crab, Paralithodes camtschaticus. Dev. Comp. Immunol..

[28] Hajji S., Younes I., Ghorbel-Bellaaj O., Hajji R., Rinaudo M., Nasri M., Jellouli K. (2014). Structural differences between chitin and chitosan extracted from three different marine sources. Int. J. Biol. Macromol..

[29] Kaya M., Seyyar O., Baran T., Erdoğan S., Kar M. (2014). A physicochemical characterization of fully acetylated chitin structure isolated from two spider species: With new surface morphology. Int. J. Biol. Macromol..

[30] Davies G.J., Knight D.P., Vollrath F. (2013). Chitin in the silk gland ducts of the spider Nephila edulis and the silkworm Bombyx mori. PLoS ONE.

[31] Synowiecki J., Al-Khateeb N.A.A.Q. (1997). Mycelia of Mucor rouxii as a source of chitin and chitosan. Food Chem..

[32] Mathur N.K., Narang C.K. (1990). Chitin and chitosan, versatile polysaccharides from marine animals. J. Chem. Educ..

[33] Zargar V., Asghari M., Dashti A. (2015). A review on chitin and chitosan polymers: Structure, chemistry, solubility, derivatives, and applications. ChemBioEng Rev..

[34] Chobot V., Kremenak J., Opletal L. (1995). Phytotherapeutic aspects of diseases of the circulatory system. 4. Chitin and chitosan. Ceska a Slovenska Farmacie: Casopis Ceske Farmaceuticke Spolecnosti a Slovenske farmaceuticke Spolecnosti.

[35] Kaya M., Asan-Ozusaglam M., Erdogan S. (2016). Comparison of antimicrobial activities of newly obtained low molecular weight scorpion chitosan and medium molecular weight commercial chitosan. J. Biosci. Bioeng..

[36] Abdou E.S., Nagy K.S., Elsabee M.Z. (2008). Extraction and characterization of chitin and chitosan from local sources. Bioresour. Technol..

[37] Mahlous M., Tahtat D., Benamer S., Khodja A.N. (2007). Gamma irradiation-aided chitin/chitosan extraction from prawn shells. Nucl. Instrum. Methods Phys. Res. Sec. B Beam Interact. Mater. At..

[38] Zhang M., Haga A., Sekiguchi H., Hirano S. (2000). Structure of insect chitin isolated from beetle larva cuticle and silkworm (*Bombyx mori*) pupa exuvia. Int. J. Biol. Macromol..

[39] Liu S., Sun J., Yu L., Zhang C., Bi J., Zhu F., Qu M., Jiang C., Yang Q. (2012). Extraction and characterization of chitin from the beetle Holotrichia parallela motschulsky. Molecules.

[40] Kaya M., Baran T. (2015). Description of a new surface morphology for chitin extracted from wings of cockroach (Periplaneta americana). Int. J. Biol. Macromol..

[41] Kaya M., Baran T., Mentes A., Asaroglu M., Sezen G., Tozak K.O. (2014). Extraction and characterization of α-chitin and chitosan from six different aquatic invertebrates. Food Biophys..

[42] Tanaka K., Katsura N., Saku T., Kasuga S. (1988). Composite texture of chitin and keratin in an animal organ, Lingula seta. Polym. J..

[43] Chaussard G., Domard A. (2004). New aspects of the extraction of chitin from squid pens. Biomacromolecules.

[44] Fan Y., Saito T., Isogai A. (2008). Preparation of chitin nanofibers from squid pen β-chitin by simple mechanical treatment under acid conditions. Biomacromolecules.

[45] Shahidi F., Abuzaytoun R. (2005). Chitin, chitosan, and co-products: Chemistry, production, applications, and health effects. Adv. Food Nutr. Res..

[46] Jang M.K., Kong B.G., Jeong Y.I., Lee C.H., Nah J.W. (2004). Physicochemical characterization of α-chitin, β-chitin, and γ-chitin separated from natural resources. J. Polym. Sci. Part A Polym. Chem..

[47] Kaya M., Baran T., Saman I., Asan Ozusaglam M., Cakmak Y.S., Menteş A. (2014). Physicochemical characterization of chitin and chitosan obtained from resting eggs of Ceriodaphnia quadrangula (Branchiopoda: Cladocera: Daphniidae). J. Crustacean Biol..

[48] Il’Ina A., Varlamov V. (2004). Hydrolysis of chitosan in lactic acid. Appl. Biochem. Microbiol..

[49] Kuroiwa T., Ichikawa S., Hiruta O., Sato S., Mukataka S. (2002). Factors affecting the composition of oligosaccharides produced in chitosan hydrolysis using immobilized chitosanases. Biotechnol. Prog..

[50] Mao S., Shuai X., Unger F., Simon M., Bi D., Kissel T. (2004). The depolymerization of chitosan: Effects on physicochemical and biological properties. Int. J. Pharm..

[51] Chen R., Chen J. (2000). Changes of polydispersity and limiting molecular weight of ultrasound-treated chitosan. Adv. Chitin Sci..

[52] Hawary D.L., Motaleb M.A., Farag H., Guirguis O.W., Elsabee M.Z. (2011). Water-soluble derivatives of chitosan as a target delivery system of 99mTc to some organs in vivo for nuclear imaging and biodistribution. J. Radioanal. Nucl. Chem..

[53] Khanjari A., Karabagias I., Kontominas M. (2013). Combined effect of N, O-carboxymethyl chitosan and oregano essential oil to extend shelf life and control Listeria monocytogenes in raw chicken meat fillets. LWT-Food Sci. Technol..

[54] Xiao B., Wan Y., Wang X., Zha Q., Liu H., Qiu Z., Zhang S. (2012). Synthesis and characterization of *N*-(2-hydroxy)propyl-3-trimethyl ammonium chitosan chloride for potential application in gene delivery. Colloids Surf. B Biointerfaces.

[55] Goy R.C., Britto D.D., Assis O.B. (2009). A review of the antimicrobial activity of chitosan. Polímeros.

[56] Liang C., Yuan F., Liu F., Wang Y., Gao Y. (2014). Structure and antimicrobial mechanism of ɛ-polylysine–chitosan conjugates through Maillard reaction. Int. J. Biol. Macromol..

[57] Reddy M.B., Arul J., Ait-Barka E., Angers P., Richard C., Castaigne F. (1998). EVect of Chitosan on Growth and Toxin Production by *Alternaria alternata* f. sp. lycopersici. Biocontrol Sci. Technol..

[58] Goy R.C., Morais S.T., Assis O.B. (2016). Evaluation of the antimicrobial activity of chitosan and its quaternized derivative on *E. coli* and *S. aureus* growth. Revista Brasileira de Farmacognosia.

[59] Jinasena D., Pathirathna P., Wickramarachchi S., Marasinghe E. Use of chitosan to control anthracnose on “Embul” banana. Proceedings of the 2011 International Conference on Asia Agriculture and Animal IPCBEE.

[60] Cheah L., Page B., Shepherd R. (1997). Chitosan coating for inhibition of sclerotinia rot of carrots. N. Z. J. Crop Hortic. Sci..

[61] Ben-Shalom N., Fallik E. (2003). Further suppression of Botrytis cinerea disease in cucumber seedlings by chitosan-copper complex as compared with chitosan alone. Phytoparasitica.

[62] Moret A., Muñoz Z., Garcés S. (2009). Control of powdery mildew on cucumber cotyledons by chitosan. J. Plant Pathol..

[63] Long L.T., Tan L.V., Boi V.N., Trung T.S. (2017). Antifungal activity of water-soluble chitosan against Colletotrichum capsici in post-harvest chili pepper. J. Food Process. Preserv..

[64] Mandal S. (2010). Induction of phenolics, lignin and key defense enzymes in eggplant (*Solanum melongena* L.) roots in response to elicitors. Afr. J. Biotechnol..

[65] Jitareerat P., Paumchai S., Kanlayanarat S., Sangchote S. (2007). Effect of chitosan on ripening, enzymatic activity, and disease development in mango (*Mangifera indica*) fruit. N. Z. J. Crop Hortic. Sci..

[66] Zeng K., Deng Y., Ming J., Deng L. (2010). Induction of disease resistance and ROS metabolism in navel oranges by chitosan. Sci. Hortic..

[67] Meng X., Yang L., Kennedy J.F., Tian S. (2010). Effects of chitosan and oligochitosan on growth of two fungal pathogens and physiological properties in pear fruit. Carbohydr. Polym..

[68] Bautista-Baños S., Hernández-López M., Bosquez-Molina E., Wilson C. (2003). Effects of chitosan and plant extracts on growth of Colletotrichum gloeosporioides, anthracnose levels and quality of papaya fruit. Crop Prot..

[69] Hassni M., El Hadrami A., Daayf F., Barka E.A., El Hadrami I. (2004). Chitosan, antifungal product against *Fusarium oxysporum* f. sp. albedinis and elicitor of defence reactions in date palm roots. Phytopathol. Mediterr..

[70] Ma Z., Yang L., Yan H., Kennedy J.F., Meng X. (2013). Chitosan and oligochitosan enhance the resistance of peach fruit to brown rot. Carbohydr. Polym..

[71] Sathiyabama M., Akila G., Einstein Charles R. (2014). Chitosan-induced defence responses in tomato plants against early blight disease caused by Alternaria solani (Ellis and Martin) Sorauer. Arch. Phytopathol. Plant Prot..

[72] Sathiyabama M., Charles R.E. (2015). Fungal cell wall polymer based nanoparticles in protection of tomato plants from wilt disease caused by *Fusarium oxysporum* f. sp. lycopersici. Carbohydr. Polym..

[73] Escudero N., Lopez-Moya F., Ghahremani Z., Zavala-Gonzalez E.A., Alaguero-Cordovilla A., Ros-Ibañez C., Lacasa A., Sorribas F.J., Lopez-Llorca L.V. (2017). Chitosan increases tomato root colonization by Pochonia chlamydosporia and their combination reduces root-knot nematode damage. Front. Plant Sci..

[74] Algam S., Xie G., Li B., Yu S., Su T., Larsen J. (2010). Effects of Paenibacillus strains and chitosan on plant growth promotion and control of Ralstonia wilt in tomato. J. Plant Pathol..

[75] Nicaise V. (2014). Crop immunity against viruses: Outcomes and future challenges. Front. Plant Sci..

[76] Faoro F. Induced Systemic Resistance against Systemic Viruses: A Feasible Approach?. https://air.unimi.it/retrieve/handle/2434/229167/299904/Faoro%20F%20_%20IOBC%20Bull.%2089.pdf.

[77] Chirkov S., Il’ina A., Surgucheva N., Letunova E., Varitsev Y.A., Tatarinova N.Y., Varlamov V. (2001). Effect of chitosan on systemic viral infection and some defense responses in potato plants. Russ. J. Plant Physiol..

[78] Bondok A. (2015). Response of Tomato Plants to Salicyli c Acid and Chitosan under Infection with Tomato mosaic virus. Am.-Eur. J. Agric. Environ. Sci..

[79] Mishra S., Jagadeesh K.S., Krishnaraj P.U., Prem S. (2014). Biocontrol of tomato leaf curl virus (ToLCV) in tomato with chitosan supplemented formulations of *Pseudomonas* sp. under field conditions. Aust. J. Crop Sci..

[80] Firmansyah D. (2017). Use of Chitosan and Plant Growth Promoting Rhizobacteria to Control Squash Mosaic Virus on Cucumber Plants. Asian J. Plant Pathol..

[81] Nagorskaya V., Reunov A., Lapshina L., Davydova V., Yermak I. (2014). Effect of chitosan on tobacco mosaic virus (TMV) accumulation, hydrolase activity, and morphological abnormalities of the viral particles in leaves of *N. tabacum* L. cv. Samsun. Virol. Sin..

[82] Jia X., Meng Q., Zeng H., Wang W., Yin H. (2016). Chitosan oligosaccharide induces resistance to Tobacco mosaic virus in Arabidopsis via the salicylic acid-mediated signalling pathway. Sci. Rep..

[83] Rabea E.I., Badawy M.E., Rogge T.M., Stevens C.V., Höfte M., Steurbaut W., Smagghe G. (2005). Insecticidal and fungicidal activity of new synthesized chitosan derivatives. Pest Manag. Sci..

[84] Sahab A., Waly A., Sabbour M., Nawar L.S. (2015). Synthesis, antifungal and insecticidal potential of Chitosan (CS)-g-poly (acrylic acid)(PAA) nanoparticles against some seed borne fungi and insects of soybean. Int. J. Chem. Tech. Res..

[85] Li Y., Qin Y., Liu S., Xing R., Yu H., Li K., Li P. (2016). Preparation, Characterization, and Insecticidal Activity of Avermectin-Grafted-Carboxymethyl Chitosan. BioMed Res. Int..

[86] Helmi A., Mohamed H.I. (2016). Biochemical and Ultrastructural Changes of Some Tomato Cultivars after Infestation with Aphis gossypii Glover (Hemiptera: Aphididae) at Qalyubiyah, Egypt. Gesunde Pflanzen.

[87] Sobhy I.S., Mandour N.S., Sarhan A.A. (2015). Tomato treatment with chemical inducers reduces the performance of Spodoptera littoralis (Lepidoptera: Noctuidae). Appl. Entomol. Zool..

[88] Razmjou J., Mohammadi M., Hassanpour M. (2011). Effect of vermicompost and cucumber cultivar on population growth attributes of the melon aphid (Hemiptera: Aphididae). J. Econ. Entomol..

[89] Carletto J., Lombaert E., Chavigny P., Brévault T., Lapchin L., Vanlerberghe-masutti F. (2009). Ecological specialization of the aphid Aphis gossypii Glover on cultivated host plants. Mol. Ecol..

[90] Tan X., Wang S., Li X., Zhang F. (2010). Optimization and application of microencapsulated artificial diet for Orius sauteri (Hemiptera: Anthocoridae). Acta Entomol. Sin..

[91] Salehi-Lisar S.Y., Bakhshayeshan-Agdam H. (2016). Drought Stress in Plants: Causes, Consequences, and Tolerance. Drought Stress Tolerance in Plants.

[92] Yang F., Hu J., Li J., Wu X., Qian Y. (2009). Chitosan enhances leaf membrane stability and antioxidant enzyme activities in apple seedlings under drought stress. Plant Growth Regul..

[93] Jiao Z., Li Y., Li J., Xu X., Li H., Lu D., Wang J. (2012). Effects of exogenous chitosan on physiological characteristics of potato seedlings under drought stress and rehydration. Potato Res..

[94] Gu L. (2011). Effects of exogenous Chitosan on physiological characteristics of phalaenopsis seedlings under draught stress. Southwest China J. Agric. Sci..

[95] Pongprayoon W., Roytrakul S., Pichayangkura R., Chadchawan S. (2013). The role of hydrogen peroxide in chitosan-induced resistance to osmotic stress in rice (*Oryza sativa* L.). Plant Growth Regul..

[96] Li Z., Zhang Y., Zhang X., Merewitz E., Peng Y., Ma X., Huang L., Yan Y. (2017). Metabolic pathways regulated by chitosan contributing to drought resistance in white clover. J. Proteome Res..

[97] Górnik K., Grzesik M., Romanowska-Duda B. (2008). The effect of chitosan on rooting of grapevine cuttings and on subsequent plant growth under drought and temperature stress. J. Fruit Ornam. Plant Res..

[98] Iriti M., Faoro F. (2008). Abscisic acid is involved in chitosan-induced resistance to tobacco necrosis virus (TNV). Plant Physiol. Biochem..

[99] Lim C.W., Baek W., Jung J., Kim J.-H., Lee S.C. (2015). Function of ABA in stomatal defense against biotic and drought stresses. Int. J. Mol. Sci..

[100] McKersie B.D., Lesheim Y. (2013). Stress and Stress Coping in Cultivated Plants.

[101] Ibrahim E.A., Ramadan W.A. (2015). Effect of zinc foliar spray alone and combined with humic acid or/and chitosan on growth, nutrient elements content and yield of dry bean (*Phaseolus vulgaris* L.) plants sown at different dates. Sci. Hortic..

[102] Ng L.M., Melcher K., Teh B.T., Xu H.E. (2014). Abscisic acid perception and signaling: Structural mechanisms and applications. Acta Pharmacol. Sin..

[103] Zhang X., Wollenweber B., Jiang D., Liu F., Zhao J. (2008). Water deficits and heat shock effects on photosynthesis of a transgenic Arabidopsis thaliana constitutively expressing ABP9, a bZIP transcription factor. J. Exp. Bot..

[104] Choi Y.-S., Kim Y.-M., Hwang O.-J., Han Y.-J., Kim S.Y., Kim J.-I. (2013). Overexpression of ArabidopsisABF3 gene confers enhanced tolerance to drought and heat stress in creeping bentgrass. Plant Biotechnol. Rep..

[105] Zagzog O.A., Gad M.M., Hafez N.K. (2017). Effect of Nano-chitosan on Vegetative Growth, Fruiting and Resistance of Malformation of Mango. Trends Hortic. Res..

[106] Reglinski T., Elmer P., Taylor J., Wood P., Hoyte S. (2010). Inhibition of Botrytis cinerea growth and suppression of botrytis bunch rot in grapes using chitosan. Plant Pathol..

[107] Meng X., Li B., Liu J., Tian S. (2008). Physiological responses and quality attributes of table grape fruit to chitosan preharvest spray and post-harvest coating during storage. Food Chem..

[108] Ferri M., Dipalo S.C., Bagni N., Tassoni A. (2011). Chitosan elicits mono-glucosylated stilbene production and release in fed-batch bioreactor cultures of grape cells. Food Chem..

[109] Scortichini M. (2014). Field efficacy of chitosan to control Pseudomonas syringae pv. actinidiae, the causal agent of kiwifruit bacterial canker. Eur. J. Plant Pathol..

[110] Gayed A.A.N.A., Shaarawi S.A.M.A., Elkhishen M.A., Elsherbini N.R.M. (2017). Pre-harvest application of calcium chloride and chitosan on fruit quality and storability of ‘Early Swelling’peach during cold storage. Ciência Agrotecnol..

[111] Giacalone G., Chiabrando V. (2013). Effect of preharvest and post-harvest application of chitosan coating on storage quality of nectarines. Acta Hortic..

[112] Jail N.G.D., Luiz C., Neto R., Di Piero R.M. (2014). High-density chitosan reduces the severity of bacterial spot and activates the defense mechanisms of tomato plants. Trop. Plant Pathol..

[113] Reddy M.B., Angers P., Castaigne F., Arul J. (2000). Chitosan effects on blackmold rot and pathogenic factors produced by Alternaria alternata in post-harvest tomatoes. J. Am. Soc. Hortic. Sci..

[114] Tsugita T., Takahashi K., Muraoka T., Fukui H. (1993). The application of chitin/chitosan for agriculture. Proceedings of the Special Session of the 7th Symposium on Chitin and Chitosan.

[115] Hirano S., Kitaura S., Sasaki N., Sakaguchi H., Sugiyama M., Hashimoto K., Tanatani A. (1996). Chitin biodegradation and wound healing in tree bark tissues. J. Polym. Environ..

[116] Kim H.-J., Chen F., Wang X., Rajapakse N.C. (2005). Effect of chitosan on the biological properties of sweet basil (*Ocimum basilicum* L.). J. Agric. Food Chem..

[117] Barka E.A., Eullaffroy P., Clément C., Vernet G. (2004). Chitosan improves development, and protects *Vitis vinifera* L. against Botrytis cinerea. Plant Cell Rep..

[118] Wanichpongpan P., Suriyachan K., Chandrkrachang S., Uragami T., Kurita K., Fukamizo T. Effects of Chitosan on the growth of Gerbera flower plant (*Gerbera jamesonii*). Proceedings of the Eighth International Chitin and Chitosan Conference and Fourth Asia Pacific Chitin and Chitosan Symposium.

[119] Chandrkrachang S. (2002). The application of chitin and chitosan in agriculture in Thailand. Adv. Chitin Sci..

[120] Xue G.-X., Gao H.-Y., Li P.-M., Zou Q. (2004). Effects of chitosan treatment on physiological and biochemical characteristics in cucumber seedlings under low temperature. J. Plant Physiol. Mol. Biol..

[121] Chookhongkha N., Sopondilok T., Photchanachai S. Effect of chitosan and chitosan nanoparticles on fungal growth and chilli seed quality. Proceedings of the International Conference on Post-harvest Pest and Disease Management in Exporting Horticultural Crops-PPDM2012 973.

[122] Ziani K., Ursúa B., Maté J.I. (2010). Application of bioactive coatings based on chitosan for artichoke seed protection. Crop Prot..

[123] Chookhongkha N., Miyagawa S., Jirakiattikul Y., Photchanachai S. Chili growth and seed productivity as affected by chitosan. Proceedings of the International Conference on Agriculture Technology and Food Sciences (ICATFS’2012).

[124] Shehata S., Fawzy Z., El-Ramady H. (2012). Response of cucumber plants to foliar application of chitosan and yeast under greenhouse conditions. Aust. J. Basic Appl. Sci..

[125] Van S.N., Minh H.D., Anh D.N. (2013). Study on chitosan nanoparticles on biophysical characteristics and growth of Robusta coffee in green house. Biocatal. Agric. Biotechnol..

[126] Wang Y., Li B., Chen X., Shi Y., Zhou Q., Qiu H., Ibrahim M., Xie G., Sun G. (2012). Effect of chitosan on seed germation, seedling growth and the clubroot control in Chinese cabbage. J. Food Agric. Environ..

[127] Kananont N., Pichyangkura R., Chanprame S., Chadchawan S., Limpanavech P. (2010). Chitosan specificity for the in vitro seed germination of two Dendrobium orchids (Asparagales: Orchidaceae). Sci. Hortic..

[128] Mondal M., Malek M., Puteh A., Ismail M., Ashrafuzzaman M., Naher L. (2012). Effect of foliar application of chitosan on growth and yield in okra. Aust. J. Crop Sci..

[129] Amini J. (2015). Induced resistance in potato plants against verticillium wilt invoked by chitosan and Acibenzolar-S-methyl. Aust. J. Crop Sci..

[130] Farouk S., Mosa A., Taha A., Ibrahim H.M., El-Gahmery A. (2011). Protective effect of humic acid and chitosan on radish (*Raphanus sativus*, L. var. sativus) plants subjected to cadmium stress. J. Stress Physiol. Biochem..

[131] Saavedra G.M., Figueroa N.E., Poblete L.A., Cherian S., Figueroa C.R. (2016). Effects of preharvest applications of methyl jasmonate and chitosan on post-harvest decay, quality and chemical attributes of Fragaria chiloensis fruit. Food Chem..

[132] Srisornkompon P., Pichyangkura R., Chadchawan S. (2014). Chitosan Increased Phenolic Compound Contents in Tea (*Camellia sinensis*) Leaves by Pre-and Post-Treatments. J. Chitin Chitosan Sci..

[133] González Gómez H., Ramírez Godina F., Ortega Ortiz H., Benavides Mendoza A., Robledo Torres V., Cabrera De la Fuente M. (2017). Use of chitosan-PVA hydrogels with copper nanoparticles to improve the growth of grafted watermelon. Molecules.

[134] Abd-Alla M., Wafaa M. (2010). New safe methods for controlling anthracnose disease of mango (*Mangifera indica* L.) fruits caused by Colletotrichum gloeosporioides (Penz.). J. Am. Sci..

[135] Abbasi N.A., Iqbal Z., Maqbool M., Hafiz I.A. (2009). Post-harvest quality of mango (*Mangifera indica* L.) fruit as affected by chitosan coating. Pak. J. Bot.

[136] Zhu X., Wang Q., Cao J., Jiang W. (2008). Effects of chitosan coating on post-harvest quality of mango (*Mangifera indica* L. cv. Tainong) fruits. J. Food Process. Preserv..

[137] Abdel Fattah A., Ashoush I., Alnashi B. (2016). Effect of Chitosan Edible Coating on Quality Attributes of Pomegranate Arils During Cold Storage. J. Food Dairy Sci. Mansoura Univ..

[138] Ghasemnezhad M., Zareh S., Rassa M., Sajedi R.H. (2013). Effect of chitosan coating on maintenance of aril quality, microbial population and PPO activity of pomegranate (*Punica granatum* L. cv. Tarom) at cold storage temperature. J. Sci. Food Agric..

[139] Petriccione M., De Sanctis F., Pasquariello M.S., Mastrobuoni F., Rega P., Scortichini M., Mencarelli F. (2015). The effect of chitosan coating on the quality and nutraceutical traits of sweet cherry during post-harvest life. Food Bioprocess Technol..

[140] Petriccione M., Mastrobuoni F., Pasquariello M.S., Zampella L., Nobis E., Capriolo G., Scortichini M. (2015). Effect of chitosan coating on the post-harvest quality and antioxidant enzyme system response of strawberry fruit during cold storage. Foods.

[141] Plainsirichai M., Leelaphatthanapanich S., Wongsachai N. (2014). Effect of chitosan on the quality of rose apples (*Syzygium agueum* Alston) cv. Tabtim Chan stored at an ambient temperature. APCBEE Procedia.

[142] Ghasemnezhad M., Shiri M. (2010). Effect of chitosan coatings on some quality indices of apricot (*Prunus armeniaca* L.) during cold storage. Casp. J. Environ. Sci..

[143] Suseno N., Savitri E., Sapei L., Padmawijaya K.S. (2014). Improving shelf-life of cavendish banana using chitosan edible coating. Procedia Chem..

[144] El Guilli M., Hamza A., Clément C., Ibriz M., Ait Barka E. (2016). Effectiveness of post-harvest treatment with chitosan to control citrus green mold. Agriculture.

[145] Drevinskas T., Naujokaitytė G., Maruška A., Kaya M., Sargin I., Daubaras R., Česonienė L. (2017). Effect of molecular weight of chitosan on the shelf life and other quality parameters of three different cultivars of Actinidia kolomikta (kiwifruit). Carbohydr. Polym..

[146] García M., Casariego A., Diaz R., Roblejo L. (2014). Effect of edible chitosan/zeolite coating on tomatoes quality during refrigerated storage. Emirates J. Food Agric..

[147] Wójcik W., Zlotek U. (2008). Use of chitosan film coatings in the storage of carrots (*Daucus carota*). Prog. Chem. Appl. Chitin Deriv..

[148] Moreira M.D.R., Ponce A., Ansorena R., Roura S.I. (2011). Effectiveness of edible coatings combined with mild heat shocks on microbial spoilage and sensory quality of fresh cut broccoli (*Brassica oleracea* L.). J. Food Sci..

[149] Carvalho R.L., Cabral M.F., Germano T.A., de Carvalho W.M., Brasil I.M., Gallão M.I., Moura C.F.H., Lopes M.M.A., de Miranda M.R.A. (2016). Chitosan coating with trans-cinnamaldehyde improves structural integrity and antioxidant metabolism of fresh-cut melon. Post-harvest Biol. Technol..

[150] Zhang Y., Zhang M., Yang H. (2015). Post-harvest chitosan-g-salicylic acid application alleviates chilling injury and preserves cucumber fruit quality during cold storage. Food Chem..

[151] Hadwiger L., Kendra D., Fristensky B., Wagoner W. (1986). Chitosan both activates genes in plants and inhibits RNA synthesis in fungi. Chitin in Nature and Technology.

[152] Limpanavech P., Chaiyasuta S., Vongpromek R., Pichyangkura R., Khunwasi C., Chadchawan S., Lotrakul P., Bunjongrat R., Chaidee A., Bangyeekhun T. (2008). Chitosan effects on floral production, gene expression, and anatomical changes in the Dendrobium orchid. Sci. Hortic..

[153] Hadwiger L.A. (2013). Multiple effects of chitosan on plant systems: Solid science or hype. Plant Sci..

[154] Van Loon L., Van Strien E. (1999). The families of pathogenesis-related proteins, their activities, and comparative analysis of PR-1 type proteins. Physiol. Mol. Plant Pathol..

[155] Ramkissoon A., Francis J., Bowrin V., Ramjegathesh R., Ramsubhag A., Jayaraman J. (2016). Bio-efficacy of a chitosan based elicitor on Alternaria solani and Xanthomonas vesicatoria infections in tomato under tropical conditions. Ann. Appl. Biol..

[156] Boon-Ek Y., Jitareerat P., Wongs-Aree C., Buanong M., Obsuwan K. (2013). Expression of Plant Defense Genes in Pepper Seedlings Treated with Chitosan Solution. Southeast Asia Symp. Qual. Manag. Post-harvest Syst..

[157] Landi L., Feliziani E., Romanazzi G. (2014). Expression of defense genes in strawberry fruits treated with different resistance inducers. J. Agric. Food Chem..

[158] Wang S.Y., Gao H. (2013). Effect of chitosan-based edible coating on antioxidants, antioxidant enzyme system, and post-harvest fruit quality of strawberries (Fragaria x aranassa Duch.). LWT-Food Sci. Technol..

[159] Zhang D., Quantick P.C. (1998). Antifungal effects of chitosan coating on fresh strawberries and raspberries during storage. J. Hortic. Sci. Biotechnol..

[160] Feliziani E., Smilanick J., Margosan D., Mansour M., Romanazzi G., Gu S., Gohil H., Ames Z.R. (2013). Preharvest fungicide, potassium sorbate, or chitosan use on quality and storage decay of table grapes. Plant Dis..

[161] Berg J.A., Appiano M., Bijsterbosch G., Visser R.G., Schouten H.J., Bai Y. (2017). Functional characterization of cucumber (*Cucumis sativus* L.) Clade V MLO genes. BMC Plant Biol..

[162] Feng H., Xia W., Shan C., Zhou T., Cai W., Zhang W. (2015). Quaternized chitosan oligomers as novel elicitors inducing protection against *B. cinerea* in Arabidopsis. Int. J. Biol. Macromol..

[163] Mejía-Teniente L., Durán-Flores F.D.D., Chapa-Oliver A.M., Torres-Pacheco I., Cruz-Hernández A., González-Chavira M.M., Ocampo-Velázquez R.V., Guevara-González R.G. (2013). Oxidative and molecular responses in *Capsicum annuum* L. after hydrogen peroxide, salicylic acid and chitosan foliar applications. Int. J. Mol. Sci..

[164] Zhao J., Sun S., Meng C., Jin Q., Fan H., Lin Y., Cai Y. (2015). Cloning and expression analysis of transcription factor gene DoWRKY1 in Dendrobium officinale. China J. Chin. Mater. Med..

[165] Liu Y., Wisniewski M., Kennedy J.F., Jiang Y., Tang J., Liu J. (2016). Chitosan and oligochitosan enhance ginger (*Zingiber officinale* Roscoe) resistance to rhizome rot caused by Fusarium oxysporum in storage. Carbohydr. Polym..

[166] Berumen-Varela G., Coronado-Partida D., Ochoa-Jiménez V., Chacón-López A., Gutiérrez-Martínez P. (2015). Effect of chitosan on the induction of disease resistance against Colletotrichum sp. in mango (*Mangifera indica* L) cv. Tommy Atkins.

[167] Lu L., Liu Y., Yang J., Azat R., Yu T., Zheng X. (2014). Quaternary chitosan oligomers enhance resistance and biocontrol efficacy of Rhodosporidium paludigenum to green mold in satsuma orange. Carbohydr. Polym..

[168] Coqueiro D.S.O., de Souza A.A., Takita M.A., Rodrigues C.M., Kishi L.T., Machado M.A. (2015). Transcriptional profile of sweet orange in response to chitosan and salicylic acid. BMC Genom..

[169] Petriccione M., Mastrobuoni F., Zampella L., Nobis E., Capriolo G., Scortichini M. (2017). Effect of chitosan treatment on strawberry allergen-related gene expression during ripening stages. J. Food Sci. Technol..

[170] Muñoz C., Hoffmann T., Escobar N.M., Ludemann F., Botella M.A., Valpuesta V., Schwab W. (2010). The strawberry fruit Fra a allergen functions in flavonoid biosynthesis. Mol. Plant.

[171] Kamalipourazad M., Sharifi M., Maivan H.Z., Behmanesh M., Chashmi N.A. (2016). Induction of aromatic amino acids and phenylpropanoid compounds in Scrophularia striata Boiss. cell culture in response to chitosan-induced oxidative stress. Plant Physiol. Biochem..

[172] Zhang D., Wang H., Hu Y., Liu Y. (2015). Chitosan controls post-harvest decay on cherry tomato fruit possibly via the mitogen-activated protein kinase signaling pathway. J. Agric. Food Chem..

[173] Bell A.A., Hubbard J.C., Liu L., Davis R.M., Subbarao K.V. (1998). Effects of chitin and chitosan on the incidence and severity of Fusarium yellows of celery. Plant Dis..

[174] Murphy J.G., Rafferty S.M., Cassells A.C. (2000). Stimulation of wild strawberry (*Fragaria vesca*) arbuscular mycorrhizas by addition of shellfish waste to the growth substrate: Interaction between mycorrhization, substrate amendment and susceptibility to red core (*Phytophthora fragariae*). Appl. Soil Ecol..

[175] Inc N.T. (2010). Efficacit´e du Lysozyme dans le Contrˆolede la Croissance des Agents Pathog`enes des Plants de Serres.

[176] O’Herlihy E.A., Duffy E.M., Cassells A.C. (2003). The effects of arbuscular mycorrhizal fungi and chitosan sprays on yield and late blight resistance in potato crops from microplants. Folia Geobot..

[177] Ohta K., Atarashi H., Shimatani Y., Matsumoto S., Asao T., Hosoki T. (2000). Effects of chitosan with or without nitrogen treatments on seedling growth in *Eustoma grandiflorum* (Raf.) Shinn. Cv. Kairyou Wakamurasaki. J. Jpn. Soc. Hortic. Sci..

[178] Spiegel Y., Kafkafi U., Pressman E. (1988). Evaluation of a protein-chitin derivative of crustacean shells as a slow-release nitrogen fertilizer on Chinese cabbage. J. Hortic. Sci..

[179] Chen Y.-E., Yuan S., Liu H.-M., Chen Z.-Y., Zhang Y.-H., Zhang H.-Y. (2016). A combination of chitosan and chemical fertilizers improves growth and disease resistance in Begonia× hiemalis Fotsch. Hortic. Environ. Biotechnol..

[180] Manjula K., Podile A. (2001). Chitin-supplemented formulations improve biocontrol and plant growth promoting efficiency of Bacillus subtilis AF 1. Can. J. Microbiol..

[181] Bakiyalakshmi S.V., Valli V., Swarnila R.D.L. (2016). Isolation and Application of Chitin and Chitosan from crab shell. Int. J. Curr. Microbiol. Appl. Sci..

[182] Silva W.O., Stamford N.P., Silva E.V., Santos C.E., Freitas A.D.S., Silva M.V. (2016). The impact of biofertilizers with diazotrophic bacteria and fungi chitosan on melon characteristics and nutrient uptake as an alternative for conventional fertilizers. Sci. Hortic..

[183] Salachna P., Wilas J., Zawadzińska A. The effect of chitosan coating of bulbs on the growth and flowering of Ornithogalum saundersiae. Proceedings of the XXIX International Horticultural Congress on Horticulture: Sustaining Lives, Livelihoods and Landscapes (IHC2014).

[184] Salachna P., Grzeszczuk M., Soból M. (2017). Effects of Chitooligosaccharide Coating Combined with Selected Ionic Polymers on the Stimulation of Ornithogalum saundersiae Growth. Molecules.

[185] Trenkel M.E. (1997). Controlled-Release and Stabilized Fertilizers in Agriculture.

[186] Savci S. (2012). Investigation of effect of chemical fertilizers on environment. Apcbee Procedia.

[187] Tamme T., Reinik M., Roasto M., Juhkam K., Tenno T., Kiis A. (2006). Nitrates and nitrites in vegetables and vegetable-based products and their intakes by the Estonian population. Food Addit. Contam..

[188] Zebarth B., Drury C., Tremblay N., Cambouris A. (2009). Opportunities for improved fertilizer nitrogen management in production of arable crops in eastern Canada: A review. Can. J. Soil Sci..

[189] Wu L., Liu M. (2008). Preparation and properties of chitosan-coated NPK compound fertilizer with controlled-release and water-retention. Carbohydr. Polym..

[190] Corradini E., De Moura M., Mattoso L. (2010). A preliminary study of the incorparation of NPK fertilizer into chitosan nanoparticles. Express Polym. Lett..

[191] Hussain M.R., Devi R.R., Maji T.K. (2012). Controlled release of urea from chitosan microspheres prepared by emulsification and cross-linking method. Iranian Polym. J..

[192] Hähndel R., Aktiengesellschaft B. (1997). Personal communication.

[193] Zhang J., Tan W., Zhang Z., Song Y., Li Q., Dong F., Guo Z. (2018). Synthesis, characterization, and the antifungal activity of chitosan derivatives containing urea groups. Int. J. Biol. Macromol..

